# Identification of an early survival prognostic gene signature for localized osteosarcoma patients

**DOI:** 10.1038/s41598-024-57527-8

**Published:** 2024-03-27

**Authors:** Tajhal D. Patel, Sandra L. Grimm, Rupa S. Kanchi, Tanmay Gandhi, Amrit Koirala, Jason T. Yustein, Cristian Coarfa

**Affiliations:** 1 Texas Children’s Cancer and Hematology Centers and The Faris D. Virani Ewing Sarcoma Center, Baylor College of Medicine, Houston, TX 770302 USA; 2 Department of Molecular and Cellular Biology, Baylor College of Medicine, Houston, TX 77030 USA; 3 Aflac Cancer and Blood Disorders Center and the Winship Cancer Institute, Emory University, 1215 Uppergate Dr., Atlanta, GA 30322 USA; 4 Dan L. Duncan Cancer Comprehensive Center, Baylor College of Medicine, Houston, TX 77030 USA; 5 Center for Precision Environmental Health, Baylor College of Medicine, One Baylor Plaza, MS:BCM305, Houston, TX 77030 USA

**Keywords:** Bone cancer, Paediatric cancer

## Abstract

Osteosarcoma is the most prevalent bone tumor in pediatric patients. Neoadjuvant chemotherapy has improved osteosarcoma patient survival, however the 5-year survival rate for localized osteosarcoma is 75% with a 30–50% recurrence rate. We, therefore, sought to identify a prognostic gene signature which could predict poor prognosis in localized osteosarcoma patients. Using the TARGET osteosarcoma transcriptomic dataset, we identified a 13-hub gene signature associated with overall survival and time to death of localized osteosarcoma patients, with the high-risk group showing a 22% and the low-risk group showing 100% overall survival. Furthermore, network analysis identified five modules of co-expressed genes that significantly correlated with survival, and identified 65 pathways enriched across 3 modules, including Hedgehog signaling, which includes 2 of the 13 genes, *IHH and GLI1*. Subsequently, we demonstrated that GLI antagonists inhibited growth of a recurrent localized PDX-derived cell line with elevated *IHH* and *GLI1* expression, but not a non-relapsed cell line with low pathway activation. Finally, we show that our signature outperforms previously reported signatures in predicting poor prognosis and death within 3 years in patients with localized osteosarcoma.

## Introduction

Osteosarcoma is the most prevalent primary bone malignancy in children and adolescents. Patients with localized disease, which constitutes about 75–80% of newly diagnosed patients, are treated with neoadjuvant chemotherapy consisting of methotrexate, doxorubicin and cisplatin (MAP) and surgical resection, and have an overall survival rate of 70–75% and an event-free survival (EFS) rate of about 60%^1–3^. Approximately 20% of patients with localized disease relapse or do not respond to the current treatment regimens. To date the most common predictive measure of localized tumor response to chemotherapy is percent tumor necrosis. Patients with localized osteosarcoma that have less than 50% tumor necrosis in response to chemotherapy (Huvos I) have extremely poor prognosis with a median EFS of only 25 months^2,4^, thus it is imperative to understand the mechanisms contributing to poor prognosis and relapse. We sought to identify genes and pathways that hinder response to current osteosarcoma treatment for localized patients, which will aid in identifying more effective targeted therapies that can overcome therapeutic resistance and act as predictive measures of non-metastatic osteosarcoma patient outcome.

**Figure 1:**
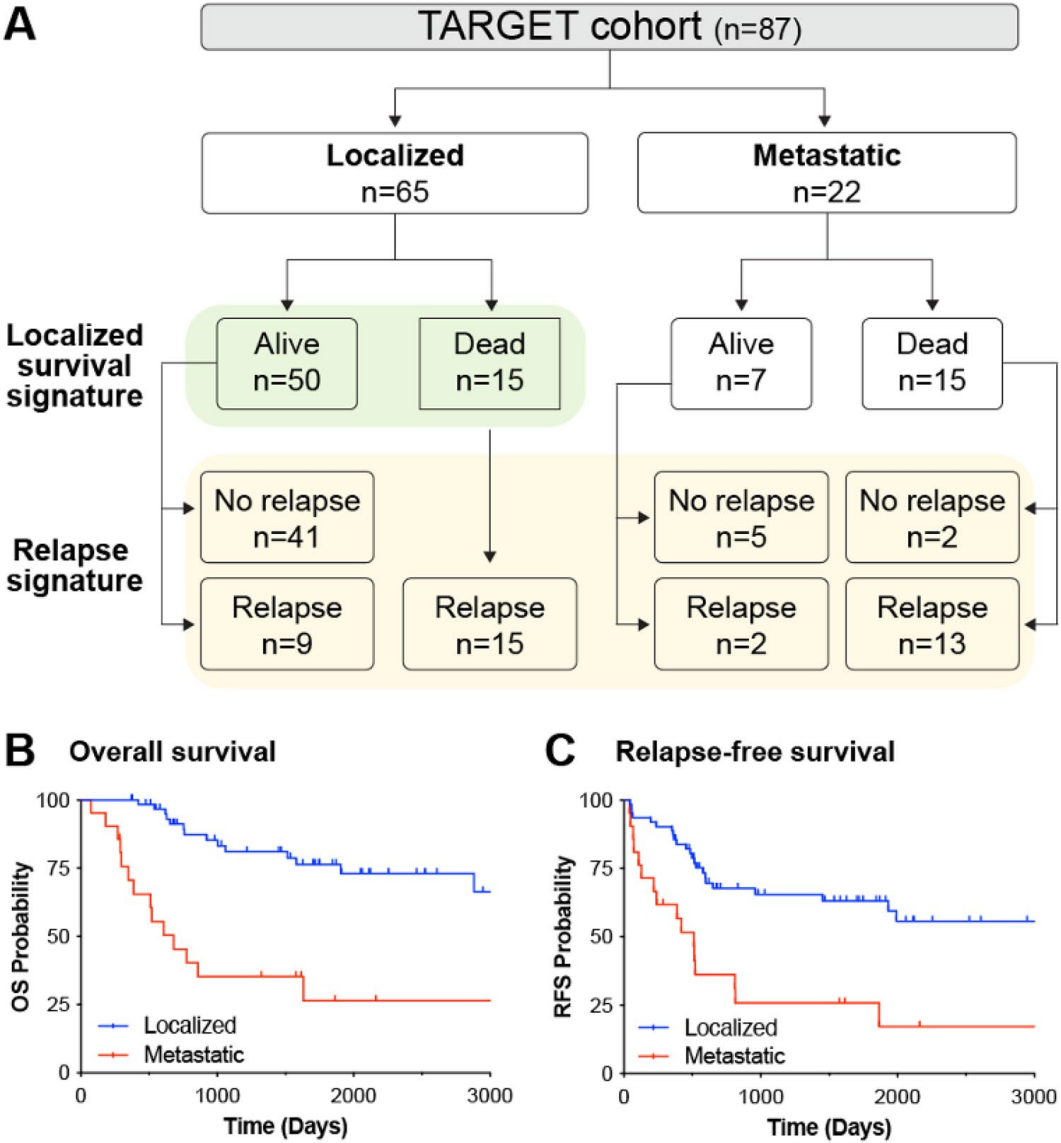
Analysis strategy using the TARGET Osteosarcoma dataset. (**A**) Patient sample numbers by non-metastatic/localized or metastatic, outcome and relapse status. Samples used for the localized survival signature are highlighted in green while samples used for the relapse signature are highlighted in yellow. (**B**) Overall survival (OS probability) and (**C**) relapse free survival (RFS probability) of TARGET patient stratified by localized (blue) or metastatic (red) tumor type.

We utilized the Therapeutically Applicable Research to Generate Effective Treatments (TARGET) database transcriptomic profiles of 87 primary tumor biopsies from osteosarcoma patients collected at the time of diagnosis to identify differentially expressed genes (DEGs) in localized osteosarcoma patients with poor prognosis (Fig. 1A). The 5-year overall survival and event-free survival of the localized patients in this cohort are 76% and 63% respectively, recapitulating previously reported data and suggesting that the results of this analysis could be informative for the broader osteosarcoma patient population (Fig. 1B,C). Using a multi-pronged analytical approach including differential gene expression, hub protein identification and Weighted Gene Correlation Network Analysis (WGCNA) we derived a 13 gene signature that predicts poor prognosis for patients with localized osteosarcoma. We further show that our signature outperforms previously reported signatures in predicting poor prognosis and death within 3 years. Our data point to the Hedgehog signaling pathway as a candidate druggable cascade to improve outcomes for high-risk non-metastatic patients. As a proof-of-concept, we were able to test two different Hedgehog pathway modulators in patient derived xenograft (PDX) cell lines and found both drugs more effective in the recurrent localized patient cell line, which had higher levels of *GLI1* and *IHH*, compared to a localized cell line derived from a therapeutically responsive, non-recurring patient. Current practices rely on post chemotherapy quantification of tumor necrosis to determine patient response to therapy; however, our study has elucidated informative molecular markers to identify at time of biopsy a high-risk localized patient population.

## Results

### Identification of 13 hub genes associated with both patient survival and relapse in localized tumors

**Figure 2:**
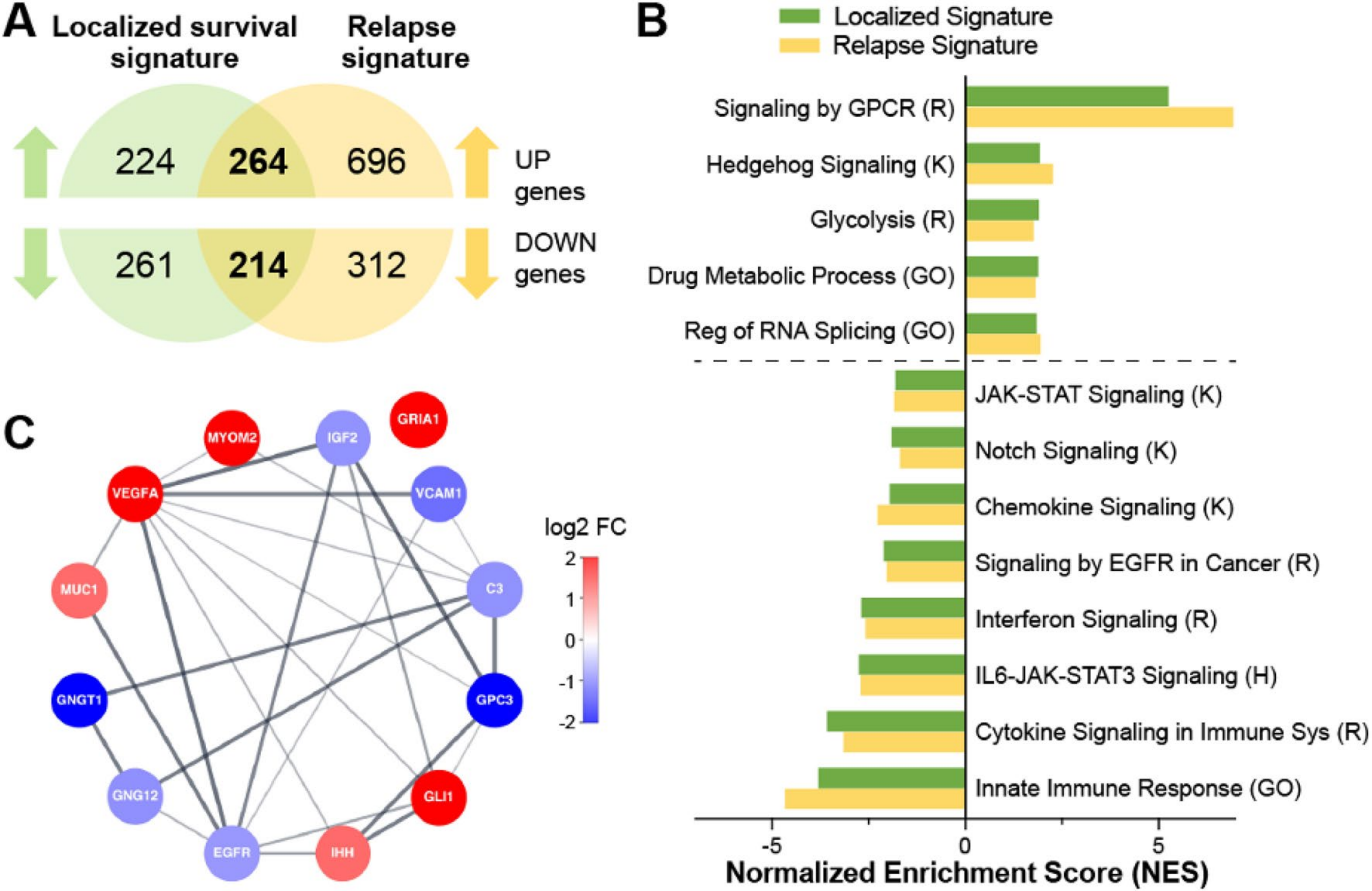
Identification of a 13 hub genes signature. (**A**) The numbers of differentially expressed increased and decreased genes from the localized survival (green) and relapse (yellow) signatures with a total of 478 genes in common between both signatures. (**B**) GSEA analysis of the localized and relapse comparisons utilizing the Gene Ontology (GO), KEGG (K), Reactome (R) and Hallmark (H) geneset compendia. Normalized enrichment scores (NES) for select genesets with an FDR < 0.25 are shown. (**C**) STRING protein interaction network for 13 hub genes; genes with higher expression in poor prognosis samples are depicted in red and genes with lower expression in poor prognosis samples are depicted in blue. Log2 FC reflects fold change of localized survival signature.

We sought to better understand the molecular mechanisms related to poor clinical outcome in patients with localized osteosarcoma. Of the 65 patients with localized tumor biopsies in the TARGET dataset (termed localized patients), 15 were deceased by the end of the study, which enabled us to compare the gene expression changes between the deceased and alive patients (Fig. 1A). Using the osteosarcoma RNAseq data from TARGET we identified 963 DEGs (488 increased and 475 decreased) with at least a 1.5 fold change and false discovery rate (FDR) < 0.05 between localized patients who died within 3 years of diagnosis and those who were alive at the end of the study (Figs. 1A and 2A highlighted in green and Supplementary Table 1). Since all the localized patients in this cohort who died had disease relapse (Fig. 1A) we also compared relapsed patients to patients who were alive and disease free at the end of the study and identified 1486 DEGs (960 increased and 526 decreased) (Figs. 1A and 2A highlighted in yellow and Supplementary Table 2). We found 478 overlapping genes (264 upregulated and 214 downregulated) between the localized survival signature and relapse signature with significant set overlap p-values of 1e-223 for up genes and 1e-220 for down genes (Fig. 2A). To identify pathways associated with the localized survival and relapse comparisons we performed Gene Set Enrichment Analysis (GSEA) utilizing the Gene Ontology (GO), Hallmark, KEGG and Reactome compendia^5–8^. Multiple enriched genesets overlapped between the two comparisons suggesting common pathways associated with both relapse and survival for localized disease patients (Fig. 2B). Specifically, there were positive enrichments for GPCR and Hedgehog signaling pathways, RNA splicing, and drug metabolic processes while there were negative enrichments for JAK/STAT, Notch, and EGFR signaling along with multiple immune related genesets (Fig. 2B).

To further elucidate genes essential to both relapse and survival for localized tumor patients we sought to identify hub proteins using betweenness centrality and radiality centrality analysis applied to the protein–protein interaction network generated from the 478 overlapping genes between the relapse and localized survival signatures (Fig. 2A)^9^. 380 of those genes were identified in the STRING database, and the corresponding protein–protein interaction network was analyzed using CytoHubba to distinguish hub genes necessary to maintain the network (Supplementary Fig. 1)^10^. Betweenness centrality computes scores for nodes/proteins based on the shortest path and importance in communicating between multiple nodes, while radiality ranks nodes central to multiple other nodes^11^. By combining the top 10 genes from each of the two scoring methods we derived 13 genes of interest: *VEGFA, EGFR, MYOM2, GNGT1, C3, MUC1, IGF2, GLI1, GNG12, GRIA1, IHH, VCAM1*, and *GPC3* (Fig. 2C). Six of these genes are increased (*MYOM2, VEGFA, MUC1, IHH, GLI1 and GRIA1*) and seven are decreased (*VCAM1, EGFR, GPC3, IGF2, GNG12, GNGT1* and *C3*) in localized patients with poor prognosis that either relapse or die due to osteosarcoma (Fig. 2C).

### 13 hub genes effectively discriminate vital status and relapse potential of non-metastatic patients with osteosarcoma

**Figure 3:**
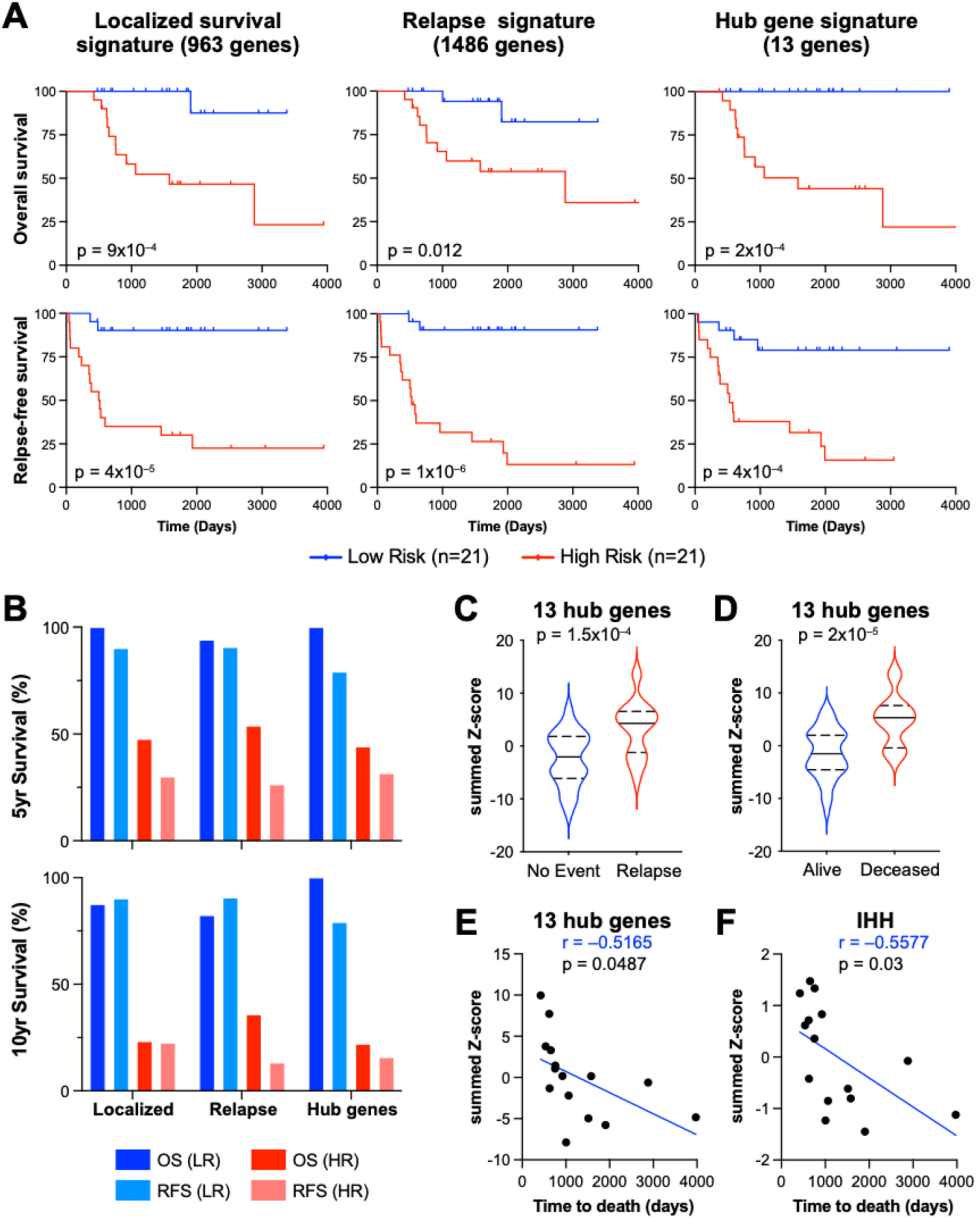
13 hub genes predict localized survival and relapse with similar results to the complete localized survival or relapse gene signatures. (**A**) Overall survival (OS) probability and relapse free survival (RFS) probability Kaplan–Meier plots; log-rank p-values were computed for localized TARGET OS patients stratified by top and bottom tertiles based on the localized survival signature, relapse signature or 13 hub genes. (**B**) Bar graph of the percent survival for high-risk (HR) and low-risk (LR) patients based on the localized survival, relapse or 13 hub gene signatures. (**C**) A 13 gene activity score was determined for each non-metastatic patient in the TARGET cohort based on expression of the 13 hub genes. The 13 gene activity is plotted based on clinical characteristics of alive/deceased or relapsed/no event (**C** and **D** respectively). (**E**) The 13 gene activity score and F. IHH mRNA levels for localized patients who died were plotted against the time to death in days. The Pearson correlation coefficient (r) and corresponding p-values are indicated.

To determine the potential of these 13 hub genes to stratify poor prognosis patients with localized osteosarcoma, we analyzed the predicted overall survival (OS) and relapse-free survival (RFS) probability using the 13 hub genes compared to using the complete localized survival or relapse signatures. Kaplan–Meier plots of the top and bottom tertiles of patients stratified based on the activity score of each gene signature (see “Methods”) was performed in the TARGET localized patient samples. Our 963 gene signature for localized osteosarcoma prognosis resulted in a 46.6% 5-year OS and 23.3% 10-year OS in the high-risk group, while the 5-year OS was 100% and 10-year OS 87.5% for the low-risk group (Fig. 3A,B). Similar to the localized signature high-risk group, the 13-hub gene signature high-risk group had 44% 5-year OS probability with 22% 10-year OS. However, the low-risk group had 100% overall 5-year and 10-year OS (Fig. 3A,B and Supplementary Fig. 2). This demonstrates that these 13 genes are as effective at predicting survival probability in patients with localized osteosarcoma as the 963 DEGs in the original localized poor prognosis signature. The relapse signature, on the other hand, predicts 13.3% 10-year RFS probability for the high-risk population and 90.7% for the low-risk group (Fig. 3A). The 13-gene signature high-risk group had 15.8% RFS with 79% in the low-risk group (Fig. 3A).

We subsequently analyzed whether any of the 13 genes identified was individually correlated with relapse or vital status (patient is deceased or alive) compared to the combined 13 genes. *GNG12, GNGT1, GRIA1, MYOM2 and VCAM1* were all significantly correlated with poor prognosis in localized patients, while low expression of *EGFR* and *GNG12* showed significant differences with regards to relapse (Supplementary Fig. 3A,B). The combined 13 genes, however, resulted in a stronger correlation with both vital status (p = 2e − 5) and relapse (p = 1.5e − 4) compared to any single gene and comparable to the full localized survival or relapse gene signatures (Fig. 3C, D and Supplementary Fig. 3A–D). This suggests that it is indeed the combined effect of all 13 genes which enhances the ability to predict relapse and prognosis for localized osteosarcoma patients in this cohort. Additionally, we correlated the combined expression score of the 13-hub genes with time to death to determine if our signature is associated with early events in localized patients. The 13 gene signature was significantly correlated with time to death in the localized patients (p = 0.048) (Fig. 3E) similarly to the 963 DEG localized survival signature but not to the 1486 DEG relapse signature (Supplementary Fig. 3E). IHH was the only individual gene to demonstrate significant correlation with time to death (Fig. 3F, p = 0.03). Taken together these data indicate that the combined 13 hub genes have prognostic association with overall survival and relapse in localized osteosarcoma patients with similar efficacy to the overall 963 localized survival DEGs, and are significantly correlated with relapse, death and time to death.

### Predictive potential of 13 hub gene signature compared to localized survival signature

**Figure 4:**
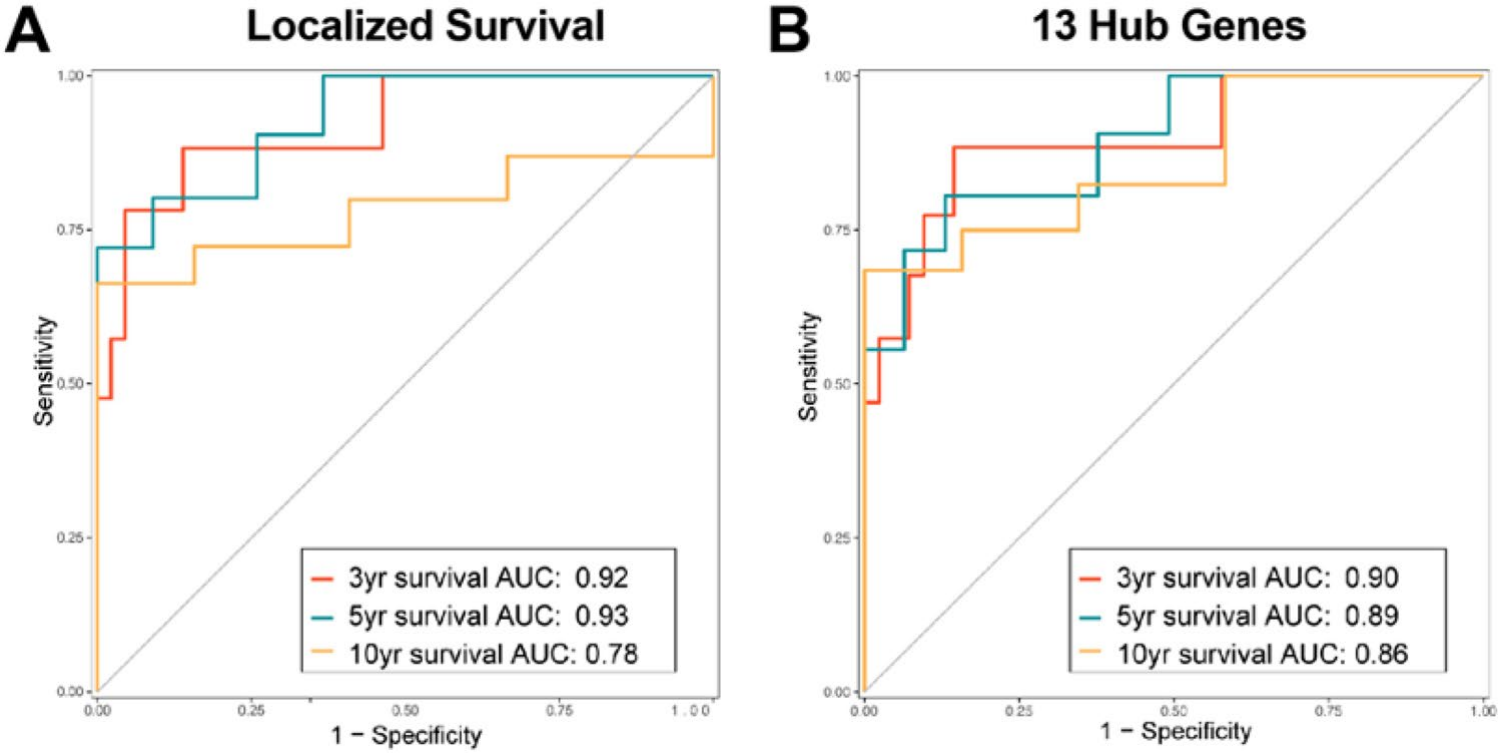
Use of time dependent ROC with the 13 hub Genes to predict localized survival and relapse. Using the localized TARGET patients with either the 963 localized gene signature or 13 hub genes time dependent ROC was performed for 3-, 5-, and 10-year vital status response for the localized survival signature (**A**) and 13 hub genes (**B**).

Due to the lack of available comprehensive clinical and transcriptomic osteosarcoma datasets containing significant numbers of non-metastatic patient samples we tested our signatures using time-dependent ROC (receiver operator curve) analysis, which estimates how well a marker measured at baseline (time = 0) determines disease status at a later time^12^. Using the timeROC package we assayed both the localized survival and 13 hub gene signatures at either 3, 5 or 10 years for vital status (deceased or alive) in non-metastatic TARGET osteosarcoma patients. At timepoints 3 and 5 years the 13 hub genes performed similar to the localized survival signature, however at the 10-year timepoint the 13 hub genes performed better with an AUC of 0.86 compared to 0.78 for the localized survival signature (Fig. 4A, B).

### Weighted Gene Correlation Network Analysis identifies 5 distinct gene clusters in patients with localized osteosarcoma

**Figure 5:**
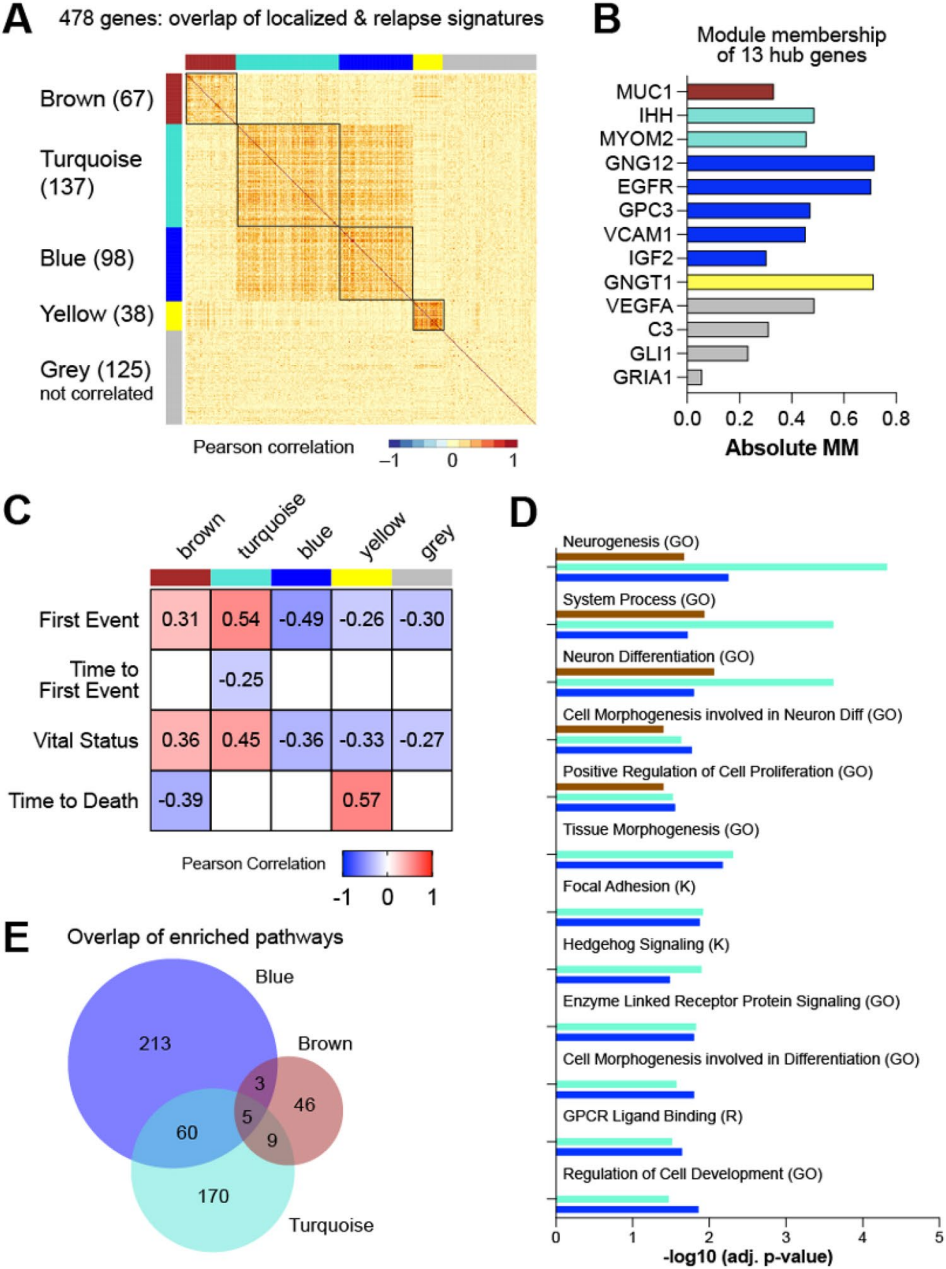
WGCNA analysis identifies 5 modules. Weighted Gene Correlation Network Analysis (WGCNA) was performed using the 478 overlapping relapse and localized poor prognosis genes resulting in 5 modules. (**A**) Pairwise Pearson correlation coefficient heatmap of the genes organized by modules, with the number of genes in each module indicated. (**B**) Module membership (MM) of each of the 13 hub genes, indicated by matching module color. (**C**) Correlation of genes within each module with TARGET clinical traits. Significant (p < 0.05) Pearson correlation coefficients are indicated. (**D**) Selected significantly enriched genesets overlapping between the brown, turquoise and blue modules; Gene Ontology (GO), KEGG (K) and REACTOME (R) significance represented as − log10(FDR). (**E**) Venn diagram of the significantly over-represented genesets within the brown, turquoise, and blue modules.

To explore whether these 13 hub genes have similar biological functions we performed Weighted Gene Correlation Network Analysis (WGCNA) on the 478 overlapping genes between the localized survival signature and relapse signature. WGCNA is a systems biology approach that identifies clusters of genes with high correlation (termed modules), which can be used to identify candidate biomarkers^13^. Five modules were identified from our analysis, including one (grey) that includes genes not correlated with genes from any module. Pairwise correlation among the 478 genes indicates that the brown and yellow modules are distinct clusters of co-expressed genes that do not correlate with genes in the other modules (Fig. 5A). In contrast, the turquoise and blue modules include genes that correlate with genes in both modules suggesting functional crosstalk (Fig. 5A). Additionally, the 13 hub genes were distributed across all five modules with 5 of the 13 genes represented in the blue module (Fig. 5B). Each module of co-expressed genes was further correlated with clinical variables in the TARGET cohort to determine whether any specific module was significantly associated with poor patient prognosis. All 5 modules were significantly correlated with first event (either death or relapse) and with vital status (deceased or alive) of non-metastatic patients, however, the blue and turquoise modules had the lowest p-values (4e − 5 and 4e − 6 respectively) indicating a greater association with poor prognosis (Fig. 5C and Supplementary Fig. 4). The turquoise module was also the most significantly correlated with vital status (p = 2e − 4).

We subsequently performed over-representation analysis (ORA) using the Hallmark, KEGG, Reactome and Gene Ontology (GO) genesets on the genes within each module to identify significantly enriched pathways. Interestingly, there were 65 genesets that overlapped between the blue and turquoise modules with 5 of those genesets also significantly enriched in the brown module (Fig. 5D). Among the overlapping genesets between the blue and turquoise modules are genesets related to development, cell differentiation, proliferation, and multiple signaling pathways including the KEGG hedgehog signaling pathway (Fig. 5E).

### Hedgehog pathway modulators affect tumor cell proliferation in recurrent osteosarcoma

**Figure 6:**
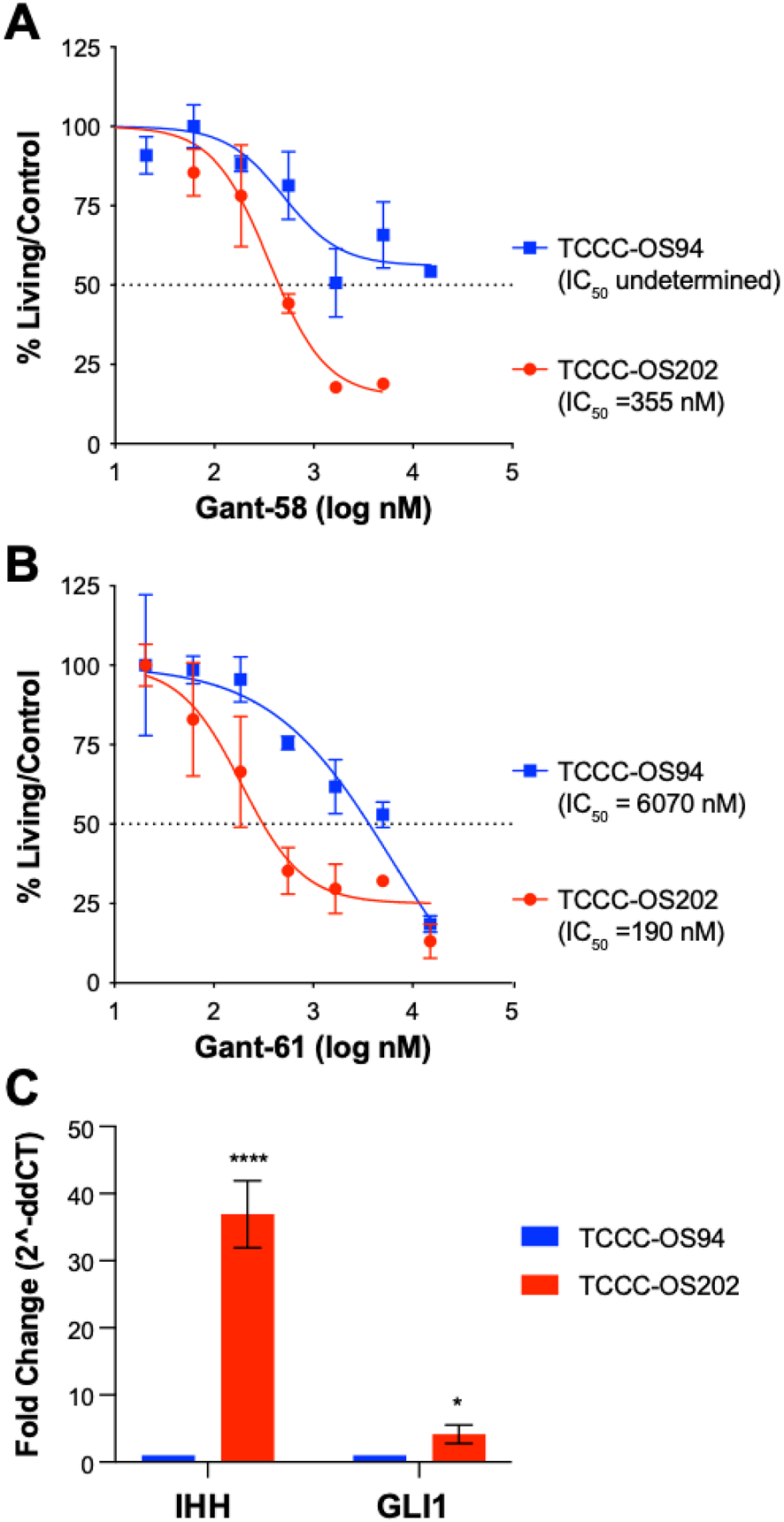
GLI antagonists growth inhibition in PDX cell lines. (**A**) The % living cells compared to control DMSO cells for increasing concentrations of GANT-58 or (**B**) GANT-61 based on CCK-8 readouts in TCCC-OS94 and TCCC-OS202 cells. (**C**) qPCR of *IHH* and *GLI1* in TCCC-OS94 and TCCC-OS202 cell lines. Values represented are fold change compared to TCCC-OS94 calculated by the ddCT method. *p < 0.05 ****p < 0.0001.

Hedgehog signaling is of particular interest since GLI1 and IHH are both upregulated in the poor prognosis signature in patients with localized, non-metastatic osteosarcoma. Additionally, we performed drug repurposing analysis using the L1000/ConnectivityMap database CMAP^14^ on the top 150 upregulated genes from the overlapping 478 gene signature to identify putative compounds capable of reversing the poor prognosis gene expression changes. Among the compounds with a reversal score greater than 90 was GANT-58 which is a GLI antagonist targeting both GLI1 and IHH (Supplementary Table 3). Therefore, we tested the effect of GANT-58 along with another GLI antagonist GANT-61 in inhibition of cell growth of 2 patient derived xenograft (PDX) cell lines. TCCC-OS94 was generated from the diagnostic biopsy in a patient with localized osteosarcoma who had greater than 90% of tumor necrosis in response to neoadjuvant MAP chemotherapy. TCCC-OS202 was derived from a local tumor recurrence in a patient with a localized disease at diagnosis who had 20% tumor necrosis in response to neoadjuvant MAP chemotherapy. As a proof-of-concept, we demonstrated that TCCC-OS202 cells had significant growth inhibition with both drugs at lower doses compared to TCCC-OS94. Moreover, TCCC-OS94 cells did not experience significant growth inhibition to GANT-58 (Fig. 6A,B). Interestingly, qPCR of IHH and GLI1 in these PDX-derived cells shows a significant increase in both IHH and GLI1 in TCCC-OS202 compared to TCCC-OS94 (Fig. 6C).

### Comparison of 13 gene hub signature to previously published OS signatures

There have been multiple published predictive signatures derived using the TARGET cohort. A 17-gene signature identified by Yang et al., was reported to better predict survival in OS patients compared to nine other comparative studies^15^. However, these ten previously published studies have combined both localized and metastatic OS patients rather than focusing on just the localized patients as in this study^15–24^; in contrast, we sought to specifically identify predictive molecular markers from upfront, diagnostic non-metastatic tumors that can help identify patients at high-risk for death/relapse. We sought to compare our 13-hub gene signature to the Yang et al. 17-gene signature in addition to the nine previously compared specifically in localized OS patients. We performed correlation of these additional signatures with time to death, overall survival and time to first event (relapse or death). Correlation to time to death performed in all osteosarcoma patient samples resulted in a Pearson correlation coefficient of − 0.37 for the Yang et.al. signature compared to − 0.38 for the 13 gene hub signature (Supplementary Fig. 5A). For overall survival and time to first event, which includes relapse, the Yang signature performed better than all others tested for all patient samples (Supplementary Fig. 5A). Multiple signatures, including our 13 gene hub signature, significantly correlated with time to first event in the localized patients only, with the Yang 17-gene signature having the lowest p-value (3.7e − 4). Additionally, the Yang 17-gene signature was the only signature to significantly correlate with overall survival for localized OS patients (Supplementary Fig. 5B). However, our 13 gene hub signature was the only signature to significantly correlate with time to death in non-metastatic OS patients specifically highlighting the association of our gene signature with poor prognosis in this particular subset of patients (Supplementary Fig. 5B).

When comparing the localized OS tertile stratified high and low-risk groups based on the Yang 17-gene signature we find that the high-risk group had a 44% 5-year OS probability which is the same as our 13-hub gene signature (Fig. 3A and Supplementary Fig. 5B). However, the 3-year OS probability of the Yang 17-gene signature was 57% for the high-risk group compared to 50% for our hub gene signature (Fig. 3A and Supplementary Fig. 5C). This suggests that while the Yang 17-gene signature does significantly correlate with overall survival, our 13-gene hub signature is better at identifying early (less than 3 year) events in the high-risk population.

To corroborate these results, we performed time-dependent ROC at 3, 5 and 10 years in localized OS patients with the Yang 17-gene signature as we did for our 13-gene hub signature. The 13-gene hub signature better predicted 3-year survival with an AUC of 0.90 compared to 0.81 for the Yang 17-gene signature (Fig. 3D and Supplementary Fig. 5C). These results were reversed for 10-year survival where the Yang signature performed better than the 13 gene hub signature with an AUC of 0.93 compared to 0.86 respectively (Fig. 3D and Supplementary Fig. 5D). These data indicate that our 13 gene hub signature better predicts early death and therefore localized patients with the poorest prognosis, while the Yang et al., 17 gene signature better predicts overall or long-term prognosis in localized OS patients.

## Discussion

In this study we sought to elucidate a gene signature specifically for upfront localized osteosarcoma patients with a high-risk for poor prognosis. While numerous studies have focused on understanding the mechanisms of metastasis, relapse and chemoresistance in osteosarcoma, there is a paucity of studies focused on identifying diagnostic genetic signatures that can help stratify localized patients into high (and low) risk populations, and subsequently help guide necessary alterations in their care and management^15–27^. The TARGET osteosarcoma dataset provides a unique opportunity to analyze this high-risk population of patients with localized osteosarcoma as it is the only current RNAseq dataset with a large number of localized patient samples which includes enough deceased patients to allow for prognostic comparisons. We identified differentially expressed genes common to relapsed osteosarcoma patients and to localized patients with poor survival outcomes. Furthermore, using a multi-pronged analysis approach including protein–protein interaction networks implemented via the STRING database and betweenness centrality and radiality centrality via CytoHubba we were able to pinpoint 13 genes central to the overall transcriptional changes in poor prognosis, localized diagnostic patient samples. This 13 gene signature, consisting of *VEGFA, EGFR, MYOM2, GNGT1, C3, MUC1, IGF2, GLI1, GNG12, GRIA1, IHH, VCAM1*, and *GPC3,* could be used effectively to potentially stratify and distinguish localized patients with high incidence of dying from their disease.

Of note is that in multiple analyses the 13-hub gene signature performed as well, and in some cases better, compared to the 963 gene localized survival signature. In fact, non-metastatic patients in the TARGET dataset stratified into the bottom tertile based on the combined 13 hub genes had 100% 10-year survival, thus identifying an ultra low-risk subpopulation of patients treated with standard osteosarcoma chemotherapeutic regimens. The 13-hub gene signature also correlated with time to death in localized patients with a similar Pearson correlation coefficient and p-value to that of the 963 gene localized signature. These results suggest that the 13 genes could act as surrogate markers of the broader gene alterations noted in the overall survival localized gene signature.

Ideally, we would validate these findings in an independent dataset, however, as previously stated there are few osteosarcoma datasets with clinical and transcriptomic data including a significant number of localized patients with poor outcome. As an alternative we performed time dependent ROC analysis to determine whether our 13-hub gene signature, which indicate alterations in upfront osteosarcoma biopsies, could predict patient survival at 3, 5 or 10 years. When compared to the 963-gene localized survival signature the 13-hub gene signature performed with similar AUC at 3 and 5 years (Fig. 4A,B). However, the 13-hub gene signature performed better than the larger 963-gene signature at predicting 10 year survival with an 86% accuracy compared to 78% for the localized signature (Fig. 4A,B). This result suggests that the 13 hub-genes measured from upfront biopsies could effectively predict patient prognosis for localized osteosarcoma patients.

There have been several signatures published based on analysis of the TARGET dataset, however these predominantly focused on mechanisms of metastasis, chemoresistance or osteosarcoma development as a whole^14–27^. The novelty of this analysis lies in the fact that the focus is on localized patients with poor outcome, with the goal of defining a gene signature that can identify, and thus stratify a high-risk population that deserves consideration for alternative therapeutic regimens due to a significant increase in succumbing to the disease within 3 years of diagnosis. To date the Yang 17-gene signature has performed the best in comparison to other published gene signatures in predicting overall patient outcome^15^. Our 13 gene hub signature, however, is more effective in identifying localized patients with poor prognosis within both 3- and 5- years of diagnosis. The identification of genes and pathways which contribute to these early events allow for targeted intervention specifically to treat this high-risk localized population.

It is possible that these 13 genes are indicative of a specific molecular mechanism and are co-regulated in a single pathway. One method of teasing out the gene–gene relationships in a network is WGCNA which can categorize genes in co-expressing modules. We identified 5 modules using the 478 overlapping relapse and localized poor prognosis genes. Interestingly, the 13 hub genes were not isolated to a single module suggesting that these genes function in distinct pathways. Two of the modules, blue and turquoise, contained genes which correlated across both modules and were most significantly correlated with poor prognosis. The turquoise module in particular had the most significant correlation to relapse and survival. This module included IHH, which is a key transducer of the hedgehog signaling pathway and is enriched for genes in the KEGG hedgehog signaling geneset. It is also interesting to note that IHH is the only gene in our 13 gene signature which individually is significantly correlated with time to death in localized TARGET osteosarcoma samples. Additionally, a second hub gene (GLI1) is also a major actor in hedgehog signaling.

Hedgehog signaling has a definitive role in normal bone development and when aberrantly activated contributes to osteosarcoma growth and metastasis^28^. Based on our current data, IHH may play a more significant role specifically in the clinical outcome of patients with non-metastatic osteosarcoma. We were able to assess this using patient derived xenograft (PDX) cell lines derived from non-metastatic patients treated with hedgehog pathway modulators (GANT-58 and GANT-61). Our data indicate that both GANT-58 and GANT-61 were more effective at growth inhibition of TCCC-OS202, which is derived from a patient with localized osteosarcoma recurrence, as compared to TCCC-OS94, which was derived from a non-relapsed, localized patient with good prognosis. Quantitative PCR of both cell lines also show higher levels of both *IHH* and *GLI1* in TCCC-OS202. While this assay was only tested on a single pair of PDX-derived cell lines, it highlights the possibility that hedgehog signaling may play a significant role and drive a negative outcome for a high-risk group of localized patients. In fact, a recent study showed GANT-61 treatment increased sensitivity to cisplatin, adriamycin, and vincristine in an in vivo chemoresistant OS cell line model^29^.

Besides *IHH* and *GLI1*, *GPC3*, which is downregulated in our poor prognosis osteosarcoma sub-group, has been shown to inhibit Hedgehog signaling in mouse development and in vitro in NIH 3T3 cells^30^. In fact, GPC3-null mice were shown to have elevated Hedgehog signaling^30^. While validation of these results in an independent cohort is still necessary, the availability of models such as the PDX cell lines used in this study allows for preliminary characterization of hedgehog pathway signaling in high-risk non-metastatic osteosarcoma. Moving forward, understanding these mechanisms could aid in fine-tuning drug combinations to provide a more targeted therapy to a high-risk subpopulation of non-metastatic osteosarcoma patients. Furthermore, while we have focused our efforts on the defining a gene signature to stratify and identify high-risk patients that will potentially require alternative, more intense therapeutic interventions, our molecular studies can potentially designate very low-risk populations of localized patients. With overall 5–10 year survival rates nearing 100%, this ultra-low subpopulation could potentially be considered for de-intensification of therapy that would reduce acute and chronic toxicities associated with the highly cytotoxic chemotherapies used to treat osteosarcoma patients. Future correlative studies incorporating these gene signatures would further validate their ability to stratify our osteosarcoma patients.

## Methods

### Data acquisition and processing

Osteosarcoma patient results were based upon data generated by the Therapeutically Applicable Research to Generate Effective Treatment (https://ocg.cancer.gov/programs/target) initiative, phs000468. Raw fastq files were downloaded from the dbGaP portal^31^. Paired-end sequencing reads were trimmed using trimGalore (https://github.com/FelixKrueger/TrimGalore), mapped using STAR^32^ against the human genome build UCSC hg38 and quantified with featureCounts^33^. Differential expression analysis was performed using the DESeq2 R package (1.28.1)^34^. P-values were adjusted with Benjamini and Hochberg’s approach for controlling the false discovery rate (FDR). Significant differentially expressed genes between the indicated comparisons were filtered based on an FDR < 0.05 and absolute fold change exceeding 1.5. P-values for the overlapping genes between two signatures was calculated using a hypergeometric test. Pathway enrichment analysis was carried out using the Gene Set Enrichment Analysis (GSEA) ^5^ (http://software.broadinstitute.org/gsea/index.jsp) software package with either Gene Ontology, KEGG, Hallmark or Reactome compendia, with significance achieved adjusted q-value < 0.25.

### Protein–protein interaction networks and betweenness and centrality analysis

Protein–protein interaction (PPI) networks were constructed in Cytoscape (version 3.9.0)^35^ using the Search Tool for the Retrieval of Interacting Genes (STRING)^10^. The hub genes of the PPI network were identified using Radiality Centrality and Betweenness Centrality categories available in the Cytohubba^9^ plugin in Cytoscape.

### Identification of co-expression modules using WGCNA

Weighted gene co-expression network analysis was performed on the 478 overlapping relapse and localized poor prognosis genes using the WGCNA (1.70) package in R^13^. Non-metastatic osteosarcoma patient sample clustering was performed to identify outliers which were removed from further analyses. Scale independence and mean connectivity were subsequently performed to determine the soft threshold for module analysis with the co-expression matrix calculated based on the optimal soft threshold of 4 and minimum module size of 30. The correlation between each module and individual clinical characteristics of the patient samples was calculated using WGCNA with significant module-trait relationships achieved for p < 0.05.

### Survival analysis and correlation

Each genes’ expression in each patient sample in the dataset was converted to a z-score. For each gene signature a patient activity score was computed by adding the z-scores of upregulated genes and subtracting the z-scores of downregulated genes in the gene signature. Patients were stratified into the top and bottom tertiles based on the signature activity score and Kaplan–Meier plots were generated in GraphPad Prism (version 9.3.1) using clinical features for the specific patients. For clinical correlations the signature activity score or individual gene z-scores were plotted against the indicated clinical features and the Pearson correlation coefficient with corresponding p-values were calculated.

### Cell lines and drug assay

Generation of osteosarcoma PDX has been describe previously^36^. PDX cell lines were grown in DMEM supplemented with 10%FBS, 100 U/ml penicillin and streptomycin, 4 mM GlutaMax (ThermoFisher) and 0.75X B-27 supplement (ThermoFisher). For drug response assays 10,000 cells per well were plated in triplicate in a 96-well plate followed by addition of various concentrations of either Doxorubicin (Sigma-Aldrich) GANT-58(Abcam) or GANT-61 (Millipore) 24 h post-plating. Cell viability was assayed with CCK-8 (Dojindo Molecular Technologies) 72 h after the addition of drug. Percent cell viability was calculated as a percentage of viable cells compared to vehicle only control wells.

### Quantitative PCR

Total RNA was extracted from cells using the Qiagen RNeasy mini kit (Qiagen) and reverse transcribed with qScript cDNA SuperMix (Quanta Biosciences) per manufacturer’s instructions. Relative quantification of gene expression was performed by quantitative RT-PCR with iTaq Universal SYBR Green supermix (Bio-Rad) on a StepOnePlus System. Reactions were performed in triplicate with each sample normalized to beta-actin and relative fold changed calculated using the 2^−ΔΔCT^ method^37^. The sequences of primers used are: GLI1 forward 5ʹ-AGCGTGAGCCTGAATCTGTG-3ʹ, GLI1 reverse 5ʹ-CAGCATGTACTGGGCTTTGAA-3ʹ, IHH forward 5ʹ-AACTCGCTGGCTATCTCGGT-3ʹ, IHH reverse 5ʹ-GCCCTCATAATGCAGGGACT-3ʹ, b-ACTIN forward 5ʹ-AGGCACCAGGGCGTGAT-3ʹ and b-ACTIN reverse 5ʹ-GCCCACATAGGAATCCTTCTGAC-3ʹ.

### Supplementary Information

Supplementary Figures.

Supplementary Tables.

## Data Availability

The results published here are in whole or part based upon data generated by the Therapeutically Applicable Research to Generate Effective Treatments (https://ocg.cancer.gov/programs/target) initiative, phs000468. The data used for this analysis are available at Genomic Data Commons (https://portal.gdc.cancer.gov).
