# Supplementary material for: Identification of an early survival prognostic gene signature for localized osteosarcoma patients
Source: Sci Rep. 2024 Mar 27;14:7327. doi: 10.1038/s41598-024-57527-8 (PMC10973371; doi:10.1038/s41598-024-57527-8)
Supplement: Supplementary file 1 — Supplementary Figures. [file 41598_2024_57527_MOESM1_ESM.pdf]

Supplementary Figure 1

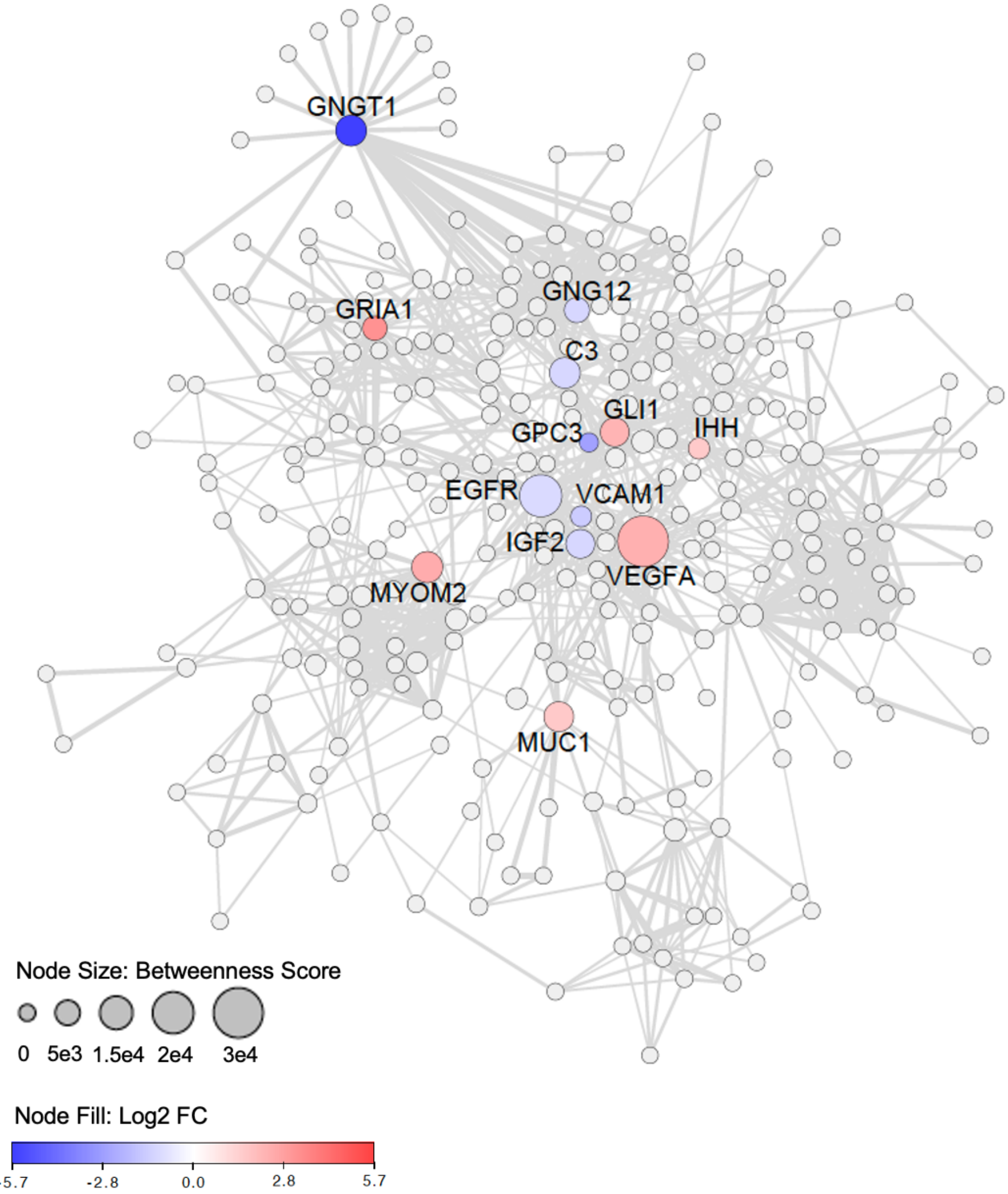

Supplementary figure 1: A protein-protein interaction network was generated using the 478 overlapping localized survival and relapse genes and the STRING database. The node size indicates betweenness centrality scores while the color indicated the log2 fold change of the 13 hub genes based on the localized survival signature.

Supplementary Figure 2

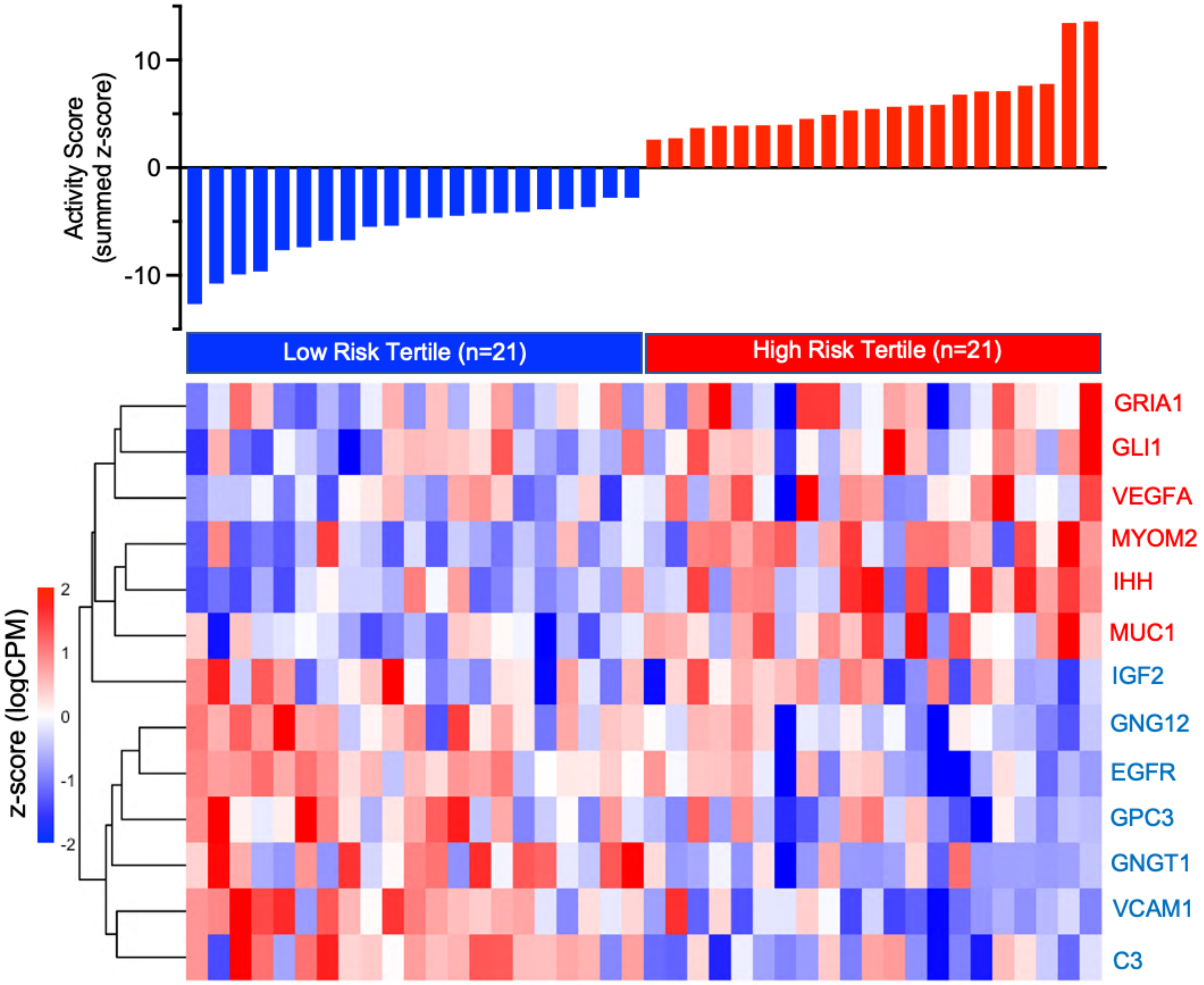

Supplementary figure 2: Identification of high-risk and low-risk tertiles in OS non-metastatic patients using the 13 hub genes signature. A bar graph of the 13 hub genes activity score is plotted for individual patients above the heatmap, with blue bars representing low risk samples and red bars denoting high risk samples. Heatmap of the expression distribution (log2 CPM) for each of the 13 hub genes. Genes labelled in red indicate genes with higher expression in poor prognosis patients while blue indicate genes with lower expression.

Supplementary Figure 3

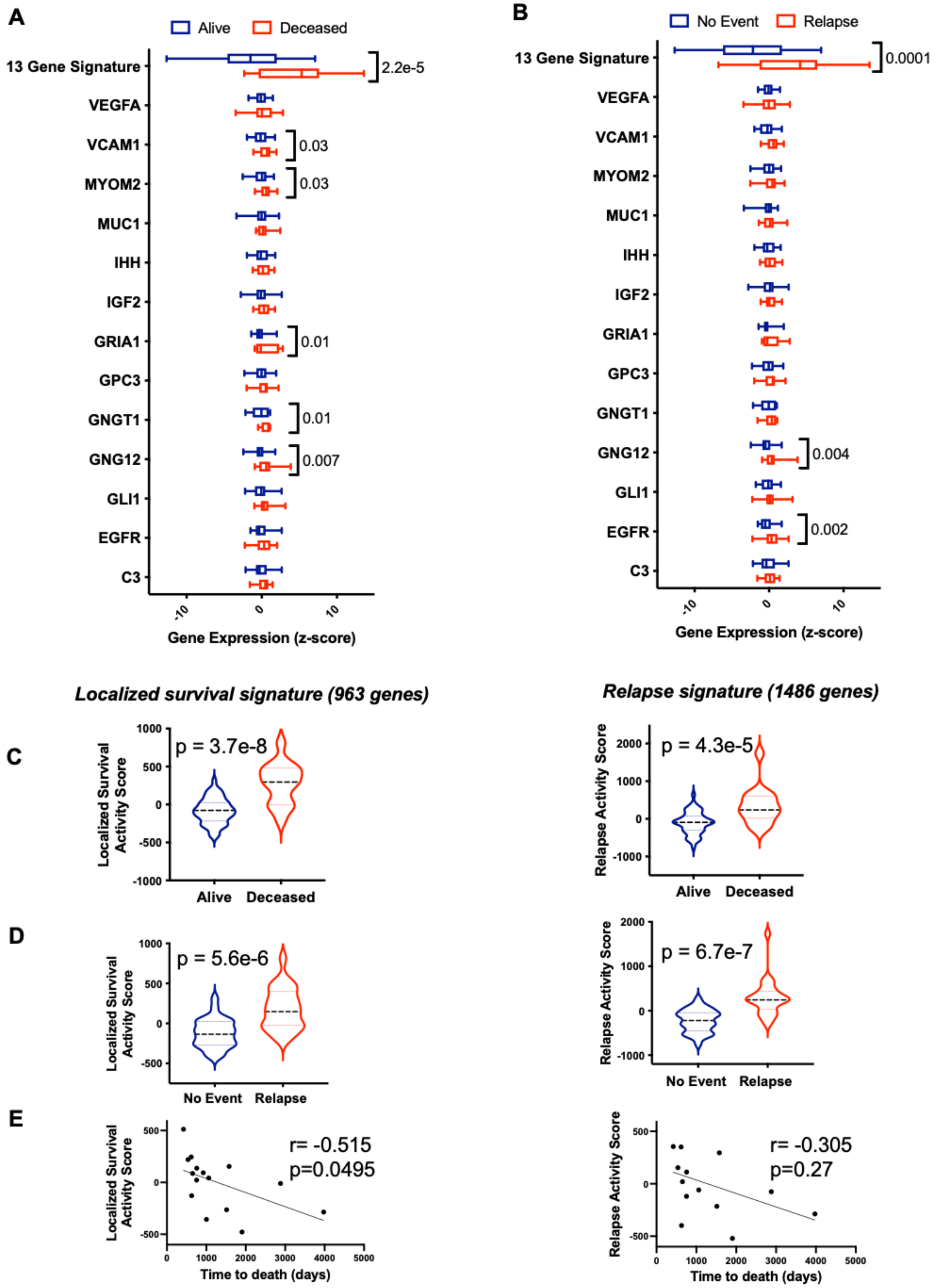

Supplementary figure 3: Association of individual genes or gene signatures with vital status and relapse. A 13 gene activity score was determined for each non-metastatic patient in the TARGET cohort. The 13 gene activity is plotted based on clinical traits of alive/deceased (A) or relapsed/no (B) event along with the activity score of each individual gene in the signature. C. The combined activity score of the localized signature and relapse signatures are plotted based on clinical characteristics of (C) alive/deceased and (D) no event/relapse. E. The localized signature activity and relapse activity score are plotted for each non-metastatic patient in the TARGET cohort against time to death. The Pearson correlation coefficient (r) and corresponding p-value are indicated. For A-D, a two-tailed unequal variance Student's t-test was used to assess significance.

Supplementary Figure 4

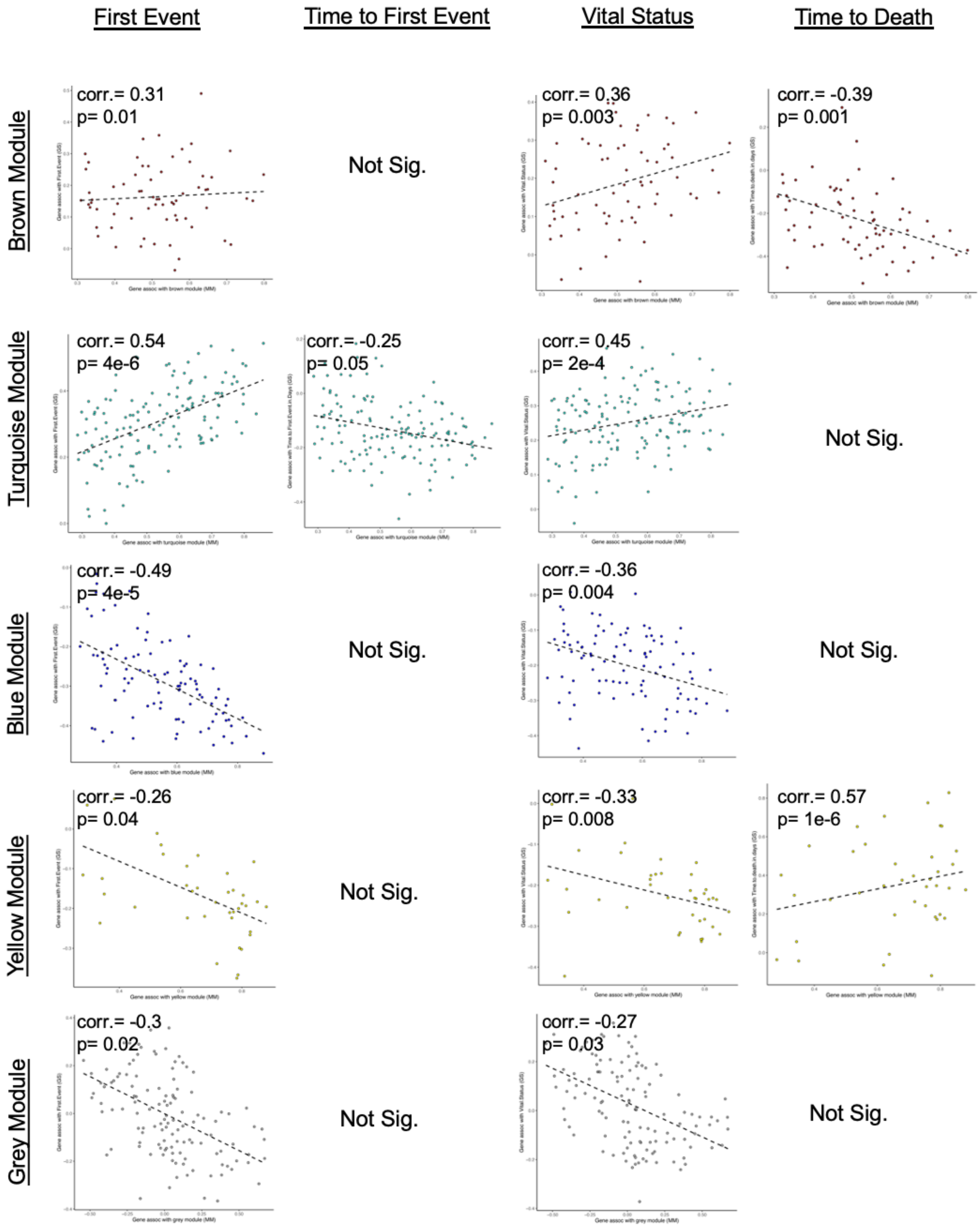

Supplementary figure 4: Correlation scatter plots of absolute module membership (MM) plotted against gene significance (GS) for each gene in the indicated module for specific clinical variables. Pearson correlation coefficients and corresponding p-values are indicated. Not Sig. denotes that the module/clinical variable correlation was not significant.

Supplementary Figure 5

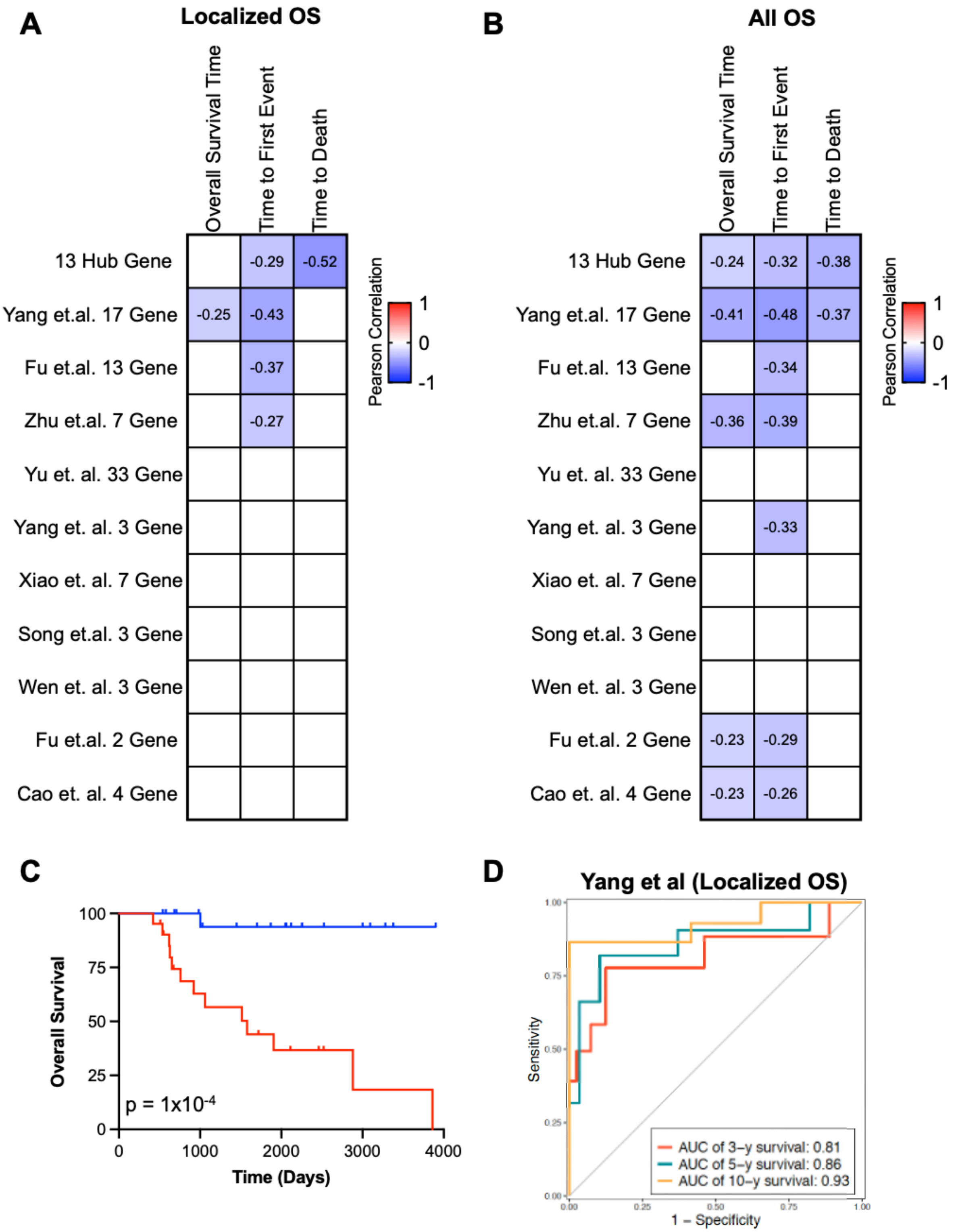

Supplementary figure 5: The 13 hub genes predict early events in localized patients while the Yang 17-gene signature predicts long-term overall survival A. Correlation of genes within each indicated signature with TARGET non-metastatic (A) or all TARGET patients (B) clinical variables. Pearson correlation coefficients are indicated for correlations which reached a significance < 0.05. C. Overall survival Kaplan-Meier plot with log-rank p-value computed for localized TARGET OS patients stratified by top and bottom tertiles based on the Yang 17-gene signature. D. Time dependent ROC was performed for 3, 5 and 10 year vital status response for the localized survival signature based on the Yang 17-gene signature.
